# Impact of proprotein convertase subtilisin/kexin type 9 (PCSK9) inhibitors on intracranial atherosclerotic plaque characteristics and low density lipoprotein-cholesterol (LDL-C) reduction: a real-world observational study

**DOI:** 10.7717/peerj.20668

**Published:** 2026-01-23

**Authors:** Zhenzhen Li, Xiaohui Li, Yiting Zhang, Jingwen Qi, Lifan Ji, Shuo Li, Mengmeng Gu, Yukai Liu, Yuqiao Zhang, Yanping Mei, Meng Wang, Junshan Zhou, Mouxiao Su, Lin Zhu, Qiwen Deng

**Affiliations:** 1 Department of Neurology, Nanjing First Hospital, Nanjing Medical University, Nanjing, China; 2 School of Basic Medicine and Clinical Pharmacy, Nanjing First Hospital, China Pharmaceutical University, Nanjing, China; 3 Department of Laboratory Medicine, Nanjing First Hospital, Nanjing Medical University, Nanjing, China; 4 Department of Neurology, School of Medicine, Mianyang Central Hospital, University of Electronic Science and Technology of China, Mianyang, China

**Keywords:** Symptomatic intracranial atherosclerotic stenosis, PCSK9 inhibitors, Plaque characteristics, Low density lipoprotein-cholesterol, High-resolution magnetic resonance imaging

## Abstract

**Background and purpose:**

The combination of PCSK9 inhibitors and moderate statin therapy effectively stabilizes intracranial atherosclerotic plaques in patients with intracranial atherosclerotic stenosis (ICAS). This study aimed to explore the effect of proprotein convertase subtilisin/kexin type 9 (PCSK9) inhibitors on plaque characteristics in patients with symptomatic ICAS (sICAS) in the anterior circulation over a 6-month follow-up.

**Methods:**

This study is a single-center, prospective, observational study, which continuously included stroke patients with sICAS in the anterior circulation. The patients were divided into two groups: the standard treatment group (atorvastatin) or the intensive treatment group (evolocumab combined with atorvastatin). The primary outcome is the change of atherosclerotic plaque characteristics over 6 months.

**Results:**

A total of 50 patients were enrolled in this study, with 34 patients ultimately included in the analysis (15 in the standard treatment group and 19 in the intensive treatment group). Both groups succeeded in reducing low-density lipoprotein-cholesterol (LDL-C) levels, and the intensive treatment group showed a more pronounced reduction (*P* < 0.001). The intensive treatment group exhibited a significant improvement in the degree of stenosis (*P* = 0.001). Notable disparities were observed between the standard treatment group and the intensive treatment group regarding percentage change of plaque length (−85.70 *vs.* −1.25%, *P* = 0.009) and plaque volume after 6 months (265.06 *vs*. 125.34 mm^3^, *P* = 0.018).

**Conclusion:**

Compared with statins alone, the utilization of PCSK9 inhibitors demonstrated a marked improvement in the progression of arteriosclerosis, effectively reducing stenosis degree, plaque length, and volume.

## Introduction

Symptomatic intracranial atherosclerotic stenosis (sICAS) refers to the narrowing of intracranial arteries caused by atherosclerosis, which can result in ischemic stroke or transient ischemic attack (TIA) within the responsible vascular supply area. It is a significant global cause to stroke incidence, with a particularly notable prevalence in Asian populations (Krasteva et al., 2020). Researches have shown that intracranial atherosclerotic stenosis (ICAS) is one of the most common causes of ischemic stroke and transient ischaemic attack (TIA) (Gutierrez et al., 2022), which accounts for 46.6% of cases (Yaghi et al., 2023). Percutaneous transluminal angioplasty and stenting (PTAS) may yield inferior outcomes compared to drug therapy alone in the management of ICAS (Hurford & Rothwell, 2020; Wang et al., 2020). Notably, findings from the SAMMPRIS trial highlighted an increased risk of stroke recurrence among ICAS patients undergoing PTAS in conjunction with intensive drug therapy, as opposed to those solely receiving drug therapy during a 12-month observational period (Chimowitz et al., 2011). The use of intensive statin therapy in patients with sICAS has become the standard of care (Turan et al., 2022). Despite aggressive medical management, individuals with ICAS still face a heightened risk of stroke recurrence, ranging from 10 to 20%, which is higher compared to other etiologies (Hurford et al., 2020; Tanaka et al., 2022; Yaghi et al., 2023). Consequently, the implementation of effective secondary prevention strategies is imperative for reducing the risk of recurrent events and mortality.

Low density lipoprotein-cholesterol (LDL-C) is a well-established risk factor for atherosclerotic cardio-cerebral diseases, and a notable reduction in LDL-C levels can markedly decrease the risk of above diseases (Giugliano et al., 2017b; Grundy et al., 2019). Statins have served as the cornerstone of lipid-lowering therapy for cardiovascular secondary prevention for a long time (Adhyaru & Jacobson, 2018; Tramacere et al., 2019). However, despite 79.6% of patients with ischemic cerebrovascular disease in China receiving lipid-lowering treatment, the compliance rate for achieving optimal LDL-C levels remains a mere 27.4% (Wang et al., 2016). Furthermore, even with the administration of maximum statin doses, some patients fail to achieve their treatment goals and may experience adverse effects, such as statin-induced muscle symptoms, as well as an increased risk of liver disease, new-onset diabetes, and cognitive decline (Adhyaru & Jacobson, 2018). Therefore, developing more effective LDL-C-lowering regimens is of critical clinical urgency for atherosclerotic patients.

The proprotein convertase subtilisin/kexin type 9 (PCSK9) is a hepatic-derived secreted protein that binds to the low density lipoprotein receptor (LDLR) and affects the internalization of low density lipoprotein (LDL), which leads to an elevation of LDL-C in the bloodstream. By binding to the PCSK9 molecule and impeding its interaction with LDLR, PCSK9 inhibitors exert a crucial role in significantly reducing LDL-C levels. This mechanism precludes receptor degradation, augmenting LDL-C uptake and thereby reducing its concentration in the bloodstream (Everett, Smith & Hiatt, 2015; Hess, Low Wang & Hiatt, 2018). Clinical trials such as FOURIER (Giugliano et al., 2017b) and ODYSSEY OUTCOMES (Goodman et al., 2023) have demonstrated a noteworthy decrease in the risk of cerebrovascular diseases, encompassing ischemic stroke and recurrent ischemic stroke, through the administration of PCSK9 inhibitors. There is no statistically significant association between PCSK9 inhibitors and the incidence of hemorrhagic stroke (Sabatine et al., 2017; Schwartz et al., 2018). Research has further indicated that PCSK9 inhibitors can stabilize and promote the regression of plaques in coronary atherosclerosis (Nicholls et al., 2022; Räber et al., 2022). Evidence suggests that a combination therapy of statins and PCSK9 inhibitors more effectively stabilizes intracranial atherosclerotic plaques in patients with ICAS compared to statins alone (Wu et al., 2023). Additionally, the adjunctive use of PCSK9 inhibitors has been shown to reduce early recurrent stroke in patients with sICAS over a 1-month follow-up period (Wu et al., 2024). High-resolution magnetic resonance imaging (HR-MRI) demonstrate that reducing LDL-C levels can stabilize intracranial atherosclerotic plaques and reduce the risk of stroke recurrence (Kim et al., 2012; Millon et al., 2013). Nevertheless, investigations examining the effect of PCSK9 inhibitors on LDL-C reduction, plaque progression and long-term prognosis in patients with sICAS remain relatively scarce. Our study bridges this gap by systematically evaluating the association between plaque characteristics and clinical outcomes during 6-month follow-up.

PCSK9 inhibitors are prospective for preventing recurrent strokes. The primary outcome of this study was to determine the effect of PCSK9 inhibitors on key characteristics related to atherosclerotic plaque progression over a 6-month period using advanced three-dimensional HRMRI (3D HRMRI) technology.

## Materials and Methods

### Study population

This study is a single-center, prospective, observational study, which continuously enrolled stroke patients with sICAS from May 2022 to June 2023 at the Nanjing First Hospital. During the screening process, all patients received secondary prevention treatment for ischemic stroke or TIA in accordance with guidelines. Patients were evaluated for eligibility based on inclusion and exclusion criteria, and after clinical advice and patient preference, they were allocated into two distinct groups: the standard treatment group, which received atorvastatin at a dosage of 40 mg, and the intensive treatment group, which received not only atorvastatin at 40 mg but also subcutaneous injections of PCSK9 inhibitor evolocumab at a dosage of 140 mg every two weeks. Both groups adhered to the medication regimen for a duration of 6 months. In case of intolerance to the dose of atorvastatin after randomization, alternative statins or other medications may be considered. The study has been approved by the Ethics Committee of Nanjing First Hospital (Ethics No. KY20220518-04-KS-01). All participants provided informed consent before enrollment, and informed consent was obtained. All subjects received standard treatments in specialized stroke units according to the guidelines of stroke prevention in sICAS (Turan et al., 2022).

### Inclusion and exclusion criteria

The following inclusion criteria were applied: (1) individuals age between 18 and 80 years old; (2) patients with mild ischemic stroke within 14 days of onset, presenting with modified Rankin Scale (mRS) score of ≤ 2; (3) individuals with moderate to severe (≥50%) symptomatic intracranial atherosclerotic stenosis in the anterior circulation, including the internal carotid artery (C6-7 segments) and the middle cerebral artery; (4) confirmation of arterial atherosclerosis through 3D High-Resolution MRI (HRMRI), with accessible images suitable for analysis; (5) baseline LDL-C > 70 mg/dl (1.8mmol/L). Patients were excluded if they met any of the following criteria: (1) contraindications to statin drugs; (2) active bleeding or significant bleeding tendencies; (3) severe ipsilateral extracranial atherosclerotic stenosis (≥50%) of the responsible artery; (4) cardiogenic embolism, such as atrial fibrillation, and left atrial or ventricular thrombus on echocardiography; (5) severe heart, lung, kidney dysfunction, tumors, immune system disorders, or other malignant diseases; (6) uncontrolled severe diabetes and hypertension; (7) non-atherosclerotic intracranial arterial stenosis, including arterial dissection; moyamoya disease; systemic vasculitis and primary central nervous system vasculitis; herpes zoster or other viral vasculitis; neurosyphilis and other intracranial infections; radiation-induced vasculopathy; fibromuscular dysplasia; sickle cell disease; neurofibromatosis; reversible cerebral vasoconstriction syndrome; postpartum vasculopathy; suspected vasospasm; suspected recanalization after vascular occlusion; (8) received angioplasty in the ipsilateral intracranial and/or extracranial arteries within 30 days before enrolment or planned to undergo endovascular intervention (including intracranial stenting, endarterectomy, and thrombectomy) within 6 months after enrollment; (9) experienced any intracranial hemorrhage within 90 days prior to enrollment; (10) used PCSK9 inhibitors within 6 months before enrolment; (11) breastfeeding and pregnant women.

Participants would be discontinued or withdrawn from the study if they met the following conditions: (1) the participant or their representative requests to withdraw their informed consent; (2) the investigator considers that the serious adverse event is associated with the treatment; (3) the participant develops another serious illness during the study, such as tumors or severe liver or kidney dysfunction; (4) loss to follow-up.

### Clinical information

We collected baseline demographic information, including age and gender, medical history, relevant risk factors such as smoking or alcohol consumption, hypertension, diabetes, history of stroke or transient ischemic attack, and history of coronary artery disease, pre-enrollment mRS scores, and National Institutes of Health Stroke Scale (NIHSS) scores at the time of onset. Laboratory examinations mainly included lipid levels (total cholesterol (TC), triglyceride (TG), high density lipoprotein-cholesterol (HDL-C, LDL-C) within 24 h before enrollment and after 6 months of medication. Based on the TST research (Amarenco et al., 2020) and the subgroup analysis of the SPARCL study (Amarenco et al., 2007), LDL-C achievement is defined as reducing LDL-C to <1.8 mmol/L or to achieve a ≥ 50% in LDL-C reduction from baseline.

### HRMRI imaging data

The MRI scans were conducted using a 3.0 T MRI scanner (Ingenia, Philips Medical Systems) with a sensitivity encoding (SENSE) parallel imaging head coil. Three-dimensional (3D) time-of-flight (TOF) MR angiography was obtained with the following parameters: repetition time (TR)/echo time (TE) of 18.6/3.4 ms, field of view (FOV) of 27.2 cm × 22 cm, flip angle of 16, slice thickness of 0.35, and 336 slices. The T1-weighted imaging in the study utilized a three-dimensional variable refocusing flip angle volume isotropic turbo spin-echo acquisition (3D T1-VISTA) technique, including the following scan parameters: repetition time (TR)/echo time (TE) of 800/18 ms, echo train length (ETL) of 16, field of view (FOV) of 200 mm × 180 mm × 40 mm, slice thickness of 0.3 mm, matrix size of 332 × 302 (yielding a isotropic spatial resolution of 0.3 mm × 0.3 mm × 0.3 mm), 1–2 number of averages, parallel imaging (SENSE) factor of 2 in the phase-encoding direction, and scan time of 378 s. The parameters for the contrast-enhanced T1-VISTA images remained consistent with those of the pre-contrast T1-VISTA images.

The processing of 3D HRMRI data involved a collaboration between Nanjing First Hospital and Jing San Medical Technology Company. Researchers from Nanjing First Hospital screened the raw imaging data of the head and neck 3D HRMRI, while researchers from Jing San Medical Technology Company performed post-processing analysis of the plaque characteristics (Extended MR WorkSpace, Philips Medical Systems). Baseline and post-treatment data were collected, including the degree of stenosis, average lumen area, remodeling index, plaque length and plaque volume.

**Figure 1:**
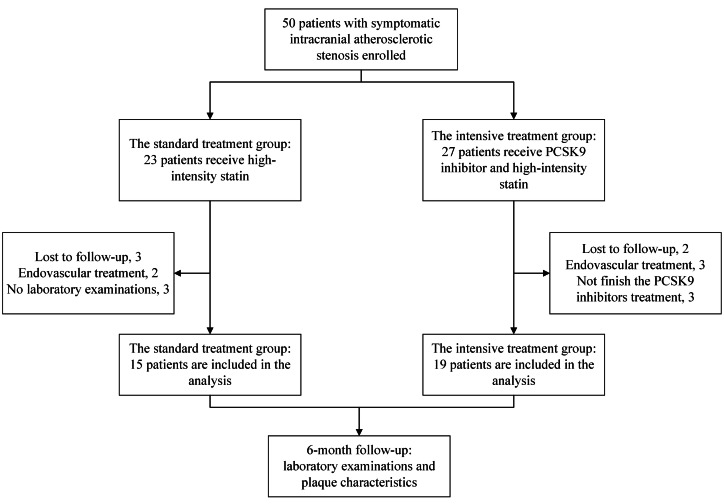
Study flow diagram. Enrollment, group allocation (standard *vs.* intensive treatment), reasons for case exclusion during follow-up, and the final number of patients included in the analysis for the study on symptomatic intracranial atherosclerotic stenosis.Abbreviations: NIHSS, National Institutes of Health Stroke Scale; mRS, modified Rankin Scale.

First, we selected the location of the lesion vessel area on the cross section. Then, the normal vessel segments contralateral or proximal to the lesion vessel area were assessed as reference values. The vessel area (VA) and lumen area (LA) at both the lesion vessel area and reference site were automatically computed by the software following manual tracing and sketching. The wall area (WA) was the difference between vessel area and lumen area. The stenosis degree was calculated as: (1- the most narrowed lumen area/reference lumen area) × 100% (Chung et al., 2020). The average lumen area is also computed automatically by the software following manual tracing and outlining. Remodeling index was calculated as the ratio of the lesion vessel area to the reference vessel area (Chung et al., 2016). The definition of atherosclerotic plaques in magnetic resonance vessel wall imaging were based on previous reports, which include focal thickening of the vessel wall with or without significant luminal stenosis. Additionally, a plaque was considered a culprit plaque when it is the only lesion in the stroke vascular territory or the most severe stenosis among multiple plaques in the same stroke vascular territory (Qiao et al., 2014). The plaque length was computed as the number of visible plaque layers × the thickness of each layer. The plaque volume was calculated as: the volume surrounded by the outer canal wall of the lesion vessel segment—the volume of the lumen. According to the change in the degree of stenosis before and after treatment (change in stenosis degree = stenosis degree before treatment—stenosis degree after treatment), it is categorized as improvement (>0) or aggravation (<0). The flowchart of this study’s participants is shown in Fig. 1.

The primary outcome of the study is the change in plaque-related characteristics during the 6-month follow-up period. The secondary outcome includes variations in LDL-C levels and achievement rates of LDL-C targets, as well as the probability of adverse events and the recurrence of ischemic cerebrovascular events in the responsible intracranial vessels. Adverse events are defined as incidents that transpire during the treatment period, encompassing cerebral hemorrhage, liver function impairment, myalgia, injection site reactions, gastrointestinal reactions, and the risk of new-onset diabetes.

### Statistical analysis

All statistical computations were performed with SPSS software (version 27.0; IBM Corp., Armonk, NY, USA). Normally distributed continuous variables were expressed as means accompanied by standard deviations, while non-normally distributed data were reported using median values with interquartile ranges. Normality was jointly determined by Shapiro–Wilk test (with *P* < 0.05 indicating rejection of the normality hypothesis) and evaluation of linear trends in Q-Q plots. For between-group comparisons of quantitative measures, either parametric *t*-tests or nonparametric Mann–Whitney tests were employed as appropriate. Frequency data were displayed as counts (percentages) and statistically evaluated using either *χ*^2^ tests or Fisher’s exact tests, depending on sample size considerations. Given the established benefits of PCSK9 inhibitors per prior studies, we used a one-tailed *P*-value for statistical testing. A one-sided *p* value of less than 0.025 (testing for superiority) was deemed statistically significant.

## Results

### Baseline information

From May 2022 to June 2023, a total of 50 patients meeting the inclusion and exclusion criteria were recruited from Nanjing First Hospital for this study. Based on clinical recommendations and patient preferences, the participants were divided into two groups: the standard treatment group with 23 cases, and the intensive treatment group with 27 cases. Some patients were excluded from the study due to reasons such as loss to follow-up, endovascular procedures performed on enrolled patients without the researcher’s awareness, and incomplete laboratory examinations. Ultimately, a total of 34 patients (the standard treatment group: 15 cases, the intensive treatment group:19 cases) were included in the analysis (Fig. 1).

The average age of the participants was 58.94 ± 10.80 years, and 24 (70.60%) of them was male. The narrowed arteries involved 88.20% of the middle cerebral arteries and 11.80% of the intracranial segments of the internal carotid arteries. The patient cohort primarily comprised individuals with mild strokes, indicated by a median NIHSS score of 2 (1–3) at onset, and a median mRS score of 0 (0–1) before enrollment. There were no statistically significant differences between the two groups at baseline concerning gender, age, medical history (hypertension, diabetes, coronary heart disease), smoking and drinking history, glycosylated hemoglobin levels, location and degree of stenosis, pre-enrollment mRS scores, and onset NIHSS scores (*P* > 0.05) (Table 1).

**Table 1:** Baseline demographics of the patients. The average age of the particip ants was 58.94 ± 10.80 years, and 24 (70.60%) of them was male. The narrowed arteries involved 88.20% of the middle cerebral arteries and 11.8% of the intracranial segments of the internal carotid arteries. The patient cohort primarily comprised individuals with mild strokes, indicated by a median NIHSS score of 2 (1–3) at onset, and a median mRS score of 0 (0–1) before enrollment. There were no statistically significant differences between the two groups at baseline concerning gender, age, medical history (hypertension, diabetes, coronary heart disease), smoking and drinking history, glycosylated hemoglobin levels, location and degree of stenosis, pre-enrollment mRS scores, and onset NIHSS scores (*P* > 0.05).

	**Total** **(*n* = 34)**	**Standard treatment group (*n* = 15)**	**Intensive treatment group (*n* = 19)**	**Mean difference or OR (95% CI)**	***P***
**Age (year), mean ± SD**	58.94 ± 10.80	62.93 ± 9.09	55.79 ± 11.22	7.14 (−0.13, 14.42)	0.054
**Male sex, n (%)**					0.276
**Male**	24 (70.60%)	9 (60.00%)	15 (78.90%)	0.76 (0.47, 1.22)	
**Female**	10 (29.40%)	6 (40.00%)	4 (21.10%)	1.90 (0.65, 5.53)	
**Stroke risk factors, n (%)**					
**Hypertension**	28 (82.40%)	11 (73.30%)	17 (89.50%)	0.82 (0.58, 1.15)	0.370
**Diabetes**	13 (38.20%)	5 (33.30%)	8 (42.10%)	0.79 (0.33, 1.93)	0.728
**Coronary artery disease**	0	0	0	0 (0, 0)	
**Smoking**	7 (20.60%)	4 (26.70%)	3 (15.80%)	1.69 (0.44, 6.42)	0.672
**Drinking**	1 (2.90%)	0	1 (4.20%)	1.06 (0.95, 1.17)	1.000
**HbA1c (%), median (IQR)**	6.05 (5.60–7.75)	6.00 (5.70–9.10)	6.20 (5.60–6.90)	0.51 (−0.64, 1.67)	0.656
**Blood pressure (mmHg), mean ± SD**				
**Systolic blood pressure**	144.06 ± 19.06	148.53 ± 16.33	140.53 ± 20.71	8.01 (−5.30, 21.32)	0.229
**Diastolic blood pressure**	88.68 ± 14.08	91.47 ± 14.83	86.47 ± 13.44	4.99 (−4.90, 14.89)	0.312
**Location of stenosis, n (%)**					0.613
**ICA**	4 (11.80%)	1 (6.70%)	3 (15.80%)	0.42 (0.05, 3.66)	
**MCA**	30 (88.20%)	14 (93.30%)	16 (84.20%)	1.11 (0.87, 1.41)	
**Stenosis degree, n (%)**					1.000
**Moderate (50%–69%)**	10 (29.40%)	4 (26.70%)	6 (31.60%)	0.84 (0.29, 2.46)	
**Severe (70%–99%)**	24 (70.60%)	11 (73.30%)	13 (68.40%)	1.07 (0.70, 1.65)	
mRS*, median (IQR)	0 (0–1)	0 (0–1)	0 (0–1)	0 (0, 0)	1.000
NIHSS*, median (IQR)	2 (1–3)	2 (1–3)	2 (1–4)	−0.79 (−2.00, 0.43)	0.732

**Notes.**

Abbreviations OR odds ratio CI confidence interval IQR interquartile range SD standard deviation HbA1c glycosylated hemoglobin ICA internal carotid artery MCA middle cerebral artery NIHSS National Institutes of Health Stroke Scale mRS modified Rankin Scale mRS* pre-enrollment mRS scores NIHSS* NIHSS scores at the time of onset

### Laboratory examinations between baseline and 6-month

Both groups exhibited reduced median LDL-C levels compared to their pre-treatment values (*P* < 0.025), indicating the effectiveness of the drug intervention. No significant differences in TC, TG, LDL-C, and HDL-C levels were observed between the two groups at baseline (*P* > 0.025). However, significant disparities in TC levels emerged between the groups at the end of the follow-up period (*P* < 0.001). Furthermore, the reduction of median LDL-C levels differed significantly for both groups (*P* < 0.001), decreasing from a baseline value of 2.44 (1.89–3.23) mmol/L (94.35 (73.09–124.90) mg/dL) to 1.78 (1.40–2.15) mmol/L (68.83 (54.14–83.14) mg/dL) in the standard treatment group, and from 2.56 (1.91–3.83) mmol/L (98.99 (73.86–148.11) mg/dL) to 1.13 (0.79–1.37) mmol/L (43.70 (30.55–52.98) mg/dL) in the intensive treatment group. The percentage change in LDL-C levels was also significantly different between the two groups (*P* < 0.001), with the intensive treatment group demonstrating a notably greater reduction compared to the standard treatment group. Regarding LDL-C compliance, 60% of patients (nine out of 15) in the standard treatment group achieved the target LDL-C level, while all 19 patients in the intensive treatment group reached the target with a 100% (19/19) compliance rate, with their LDL-C levels all below 1.8 mmol/L. Patients receiving intensive treatment achieved significantly greater LDL-C compliance compared to the standard treatment group (*P* = 0.002) (Table 2).

**Table 2:** Comparison of lipid levels between baseline and the 6-month follow-up in standard and intensive treatment groups.

	**Standard treatment group (*n* = 15)**	**Intensive treatment group (*n* = 19)**	***P***
**TC (mmol/L)**			
**Baseline, mean ± SD**	4.07 ± 0.92	4.53 ± 1.24	0.118
**Six months, mean ± SD**	3.82 ± 1.07	2.51 ± 0.70	<0.001
**Percent change (%), median (IQR)**	−10.38 (−26.84–17.28)	−49.09 (−60.00–31.90)	<0.001
**TG (mmol/L)**			
**Baseline, median (IQR)**	1.32 (1.01–2.37)	1.18 (0.84–2.61)	0.431
**Six months, median (IQR)**	1.14 (0.94–1.33)	1.27 (0.87–1.87)	0.431
**Percent change (%), median (IQR)**	−10.14 (−34.48–6.56)	−10.37 (−44.06–10.71)	0.384
**LDL (mmol/L)**			
**Baseline, median (IQR)**	2.44 (1.89–3.23)	2.56 (1.91–3.83)	0.244
**Six months, median (IQR)**	1.78 (1.40–2.15)	1.13 (0.79–1.37)	<0.001
**Percent change (%), median (IQR)**	−28.62 (−50.39–12.07)	−61.54 (−75.20–52.07)	<0.001
**Up to standard, n (%)**	9 (60%)	19 (100%)	0.002
**Not up to standard, n (%)**	6 (40%)	0 (0%)	
**HDL (mmol/L)**			
**Baseline, median (IQR)**	0.89 (0.83–1.09)	0.99 (0.76–1.24)	0.451
**Six months, median (IQR)**	1.02 (1.00–1.28)	1.04 (0.87–1.17)	0.193
**Percent change (%), median (IQR)**	19.09 (4.21–33.33)	8.16 (−1.89–27.45)	0.075

**Notes.**

Abbreviations IQR interquartile range SD standard deviation TC total cholesterol TG triglyceride LDL-C low-density lipoprotein-cholesterol HDL-C high- density lipoprotein-cholesterol

Percent change(%) = (6-month level − baseline level)/baseline level×100%.

**Figure 2:**
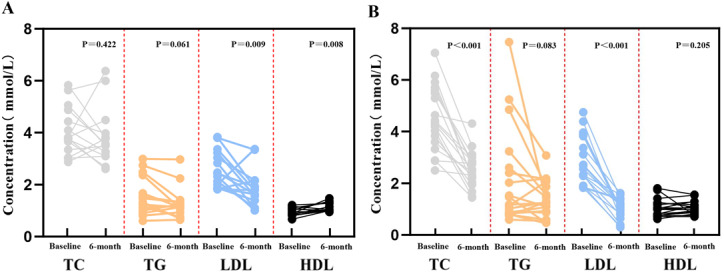
Comparison of blood lipid parameters between the two groups at baseline and 6-month follow-up. (A) Patients receiving standard treatment; (B) Patients receiving intensive treatment. Abbreviations: TC, total cholesterol; TG, triglycerides; LDL-C, low-density lipoprotein cholesterol; HDL-C, high-density lipoprotein cholesterol.

Figure 2 showed the levels of lipid parameters at baseline and after 6 months of treatment for each patient in the two groups. Compared with baseline, the level of LDL-C and HDL-C was significantly decreased after standard treatment (*P* < 0.025). Meanwhile, significant disparities in TC and LDL-C levels were shown after intensive treatment (*P* < 0.001). Moreover, it is evident that the decrease in LDL-C levels in the intensive treatment group is markedly greater than that in the standard treatment group.

### Plaque characteristics between baseline and 6-month

**Figure 3:**
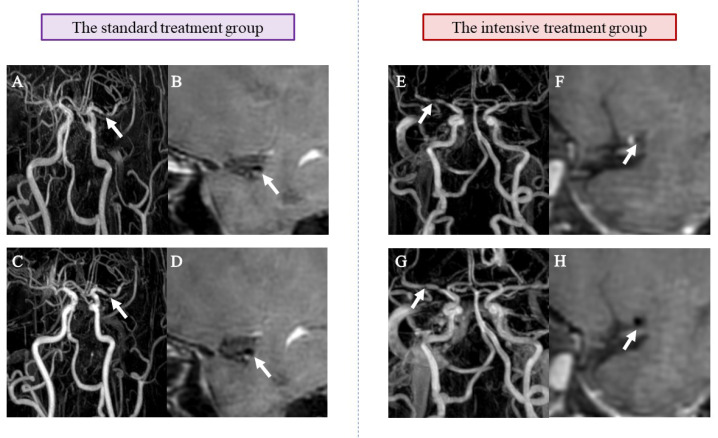
Example HRMRI images of sICAS patients in different groups at baseline and 6-month follow-up. (A) and (E) show demonstrated MRA findings consistent with atherosclerotic stenosis at baseline (arrow shows vessel stenosis); (B) and (F) show post-contrast (CE-T1W) HRMRI at baseline (arrow shows vascular secretion and plaque)); (C) and (G) show MRA results consistent with atherosclerotic stenosis at 6 months (arrow shows vessel stenosis); (D) and (H) show post-contrast (CE-T1W) HRMRI at 6 months (arrow shows vascular valves and plaques). Compared with A and B, C and D showed no significant changes in the degree of stenosis and plaque characteristics, whereas G and H showed significant improvement compared with E and F.

No significant difference was observed in baseline imaging findings between the two groups, except for the average lumen area (*P* = 0.009). Following a 6-month follow-up, the intensive treatment group exhibited improvement in the stenosis degree of the responsible artery in nine cases, manifesting in a 47.37% improvement rate and a 15.79% aggravation rate. Conversely, the standard treatment group displayed a 0% improvement rate and a 20% aggravation rate. The improvement rate for the intensive treatment group is significantly higher than that for the standard treatment group (*P* = 0.001). The intensive treatment group showed a significantly greater reduction in plaque length percentage change compared to the standard treatment group at the 6-month follow-up (*P* < 0.025). Similarly, patients in the intensive treatment group exhibited a lesser plaque volume than those in the standard treatment group after 6 months. Nevertheless, the two groups had no significant differences in the percentage change of remodeling index (*P* = 0.450) and average lumen area (*P* = 0.214) (Table 3). Example HRMRI images of sICAS patients in different groups at baseline and the 6-month follow-up are shown in Fig. 3.

**Table 3:** Comparison of plaque characteristics between baseline and 6-month follow-up in standard and intensive treatment groups. Includes changes in stenosis degree (improvement/aggravation), remodeling index, average lumen area, plaque length, and plaque volume, providing percent changes for each measure and the P-values for intergroup comparisons.

	**Standard treatment group** **(*n* = 15)**	**Intensive treatment group** (***n* = 19)**	***P***
**Stenosis degree**			
**Improvement, n (%)**	0 (0%)	9 (47.37%)	0.001
**Aggravation**, **n (%)**	3 (20%)	3 (15.79%)	0.274
**Remodeling index**			
**Baseline, median (IQR)**	0.80 (0.68–1.21)	0.69 (0.65–1.47)	0.090
**Six months, median (IQR)**	1.10 (0.83–1.37)	0.85 (0.72–1.81)	0.151
**Percent change (%), median (IQR)**	9.46 (−10.02–58.5)	9.14 (0.00–32.96)	0.450
**Average lumen area (mm** ^**2**^ **)**			
**Baseline, median (IQR)**	5.34 (3.61–10.51)	3.32 (1.76–4.04)	0.009
**Six months, median (IQR)**	3.44 (2.59–13.56)	3.74 (1.95–5.12)	0.085
**Percent change (%), median (IQR)**	−21.69 (−35.96–59.77)	11.68 (−24.83–40.32)	0.214
**Plaque length (mm)**			
**Baseline, mean ± SD**	18.32 ± 10.02	17.73 ± 6.63	0.423
**Six months, mean ± SD**	33.36 ± 28.55	20.18 ± 8.71	0.038
**Percent change (%), median (IQR)**	85.70 (−18.19–218.75)	−1.25 (−21.87–40.16)	0.009
**Plaque volume(mm** ^**3**^ **)**			
**Baseline, median (IQR)**	137.00 (79.16–395.19)	108.46 (74.07–193.30)	0.212
**Six months, median (IQR)**	265.06 (151.58–523.78)	125.34 (98.63–214.14)	0.018
**Percent change (%), median (IQR)**	75.67 (−31.13–323.45)	5.98 (−17.40–31.37)	0.057

**Notes.**

Percent change(%) = (6-month level − baseline level)/baseline level × 100%.

### Outcome events and adverse events during 6-month follow-up

During the 6-month treatment follow-up period, five cases of recurrent ischemic cerebrovascular events occurred in the standard treatment group (33.33%), whereas only one case emerged in the intensive treatment group (5.30%). There was no statistically significant disparity between the two groups (*P* = 0.033), which might be attributed to the small sample size. Notably, there were no cases of disabling stroke or death in either group. Comparisons of cerebral hemorrhage, liver function injury, muscle pain, injection site reactions, gastrointestinal reactions, and risk of new-onset diabetes showed no significant differences between the two groups (*P* > 0.025) (Table 4).

**Table 4:** Comparison of occurrence rates of outcome events and adverse events in in standard and intensive treatment groups. Rates of recurrent ischemic cerebrovascular events, modified Rankin Scale (mRS) scores, and the incidence of various adverse events (including liver injury) between the two groups during the 6-month follow-up period.

	**Standard treatment group (*n* = 15)**	**Intensive treatment group** **(*n* = 19)**	***P***
**Ischemic cerebrovascular events, n (%)**	5 (33.33%)	1 (5.30%)	0.033
mRS*	0 (0–1)	0 (0–0)	0.052
**Death**	0	0	
**Adverse events, n (%)**			
**Cerebral hemorrhage**	0	0	
**Liver function injury, n (%)**	1 (6.66%)	1 (5.30%)	1.000
**Myalgia**	0	0	
**Injection site reaction**	0	0	
**Gastrointestinal reaction**	0	0	
**Risk of new-onset diabetes**	0	0	

**Notes.**

Abbreviations mRS modified Rankin Scale mRS* mRS scores at 6 months.

## Discussion

Our study revealed that the utilization of PCSK9 inhibitors in conjunction with statins significantly reduced LDL-C levels and increased the rate of achieving LDL-C targets compared to statin monotherapy. The intensive treatment group successfully decreased LDL-C levels from 2.56 mmol/L (98.99 mg/dL) to 1.13 mmol/L (43.70 mg/dL), with all 19 patients achieving the target, resulting in a compliance rate of 100% (19/19). Furthermore, the intensive treatment group demonstrated a higher rate of stenosis degree improvement in contrast to the standard treatment group (47.37% *vs.* 0%). Moreover, the intensive treatment group exhibited more pronounced reductions in plaque volume (265.06 *vs.* 125.34 mm^3^, *P* = 0.018) and percentage change in plaque length (−85.70 *vs.* −1.25%, *P* = 0.009). Additionally, there were no significant differences between the two groups in the incidence of adverse events.

sICAS presents a notable susceptibility to early recurrent strokes, with higher plaque burden and enhancement ratio identified as independent risk factors for stroke recurrence in symptomatic intracranial atherosclerotic disease (Lv et al., 2023). Elevated LDL-C levels have been associated with the formation, volume, and burden of atherosclerotic plaques (Metzner et al., 2022; Virani et al., 2011), and are established risk factors for atherosclerotic cardiovascular and cerebrovascular diseases (Giugliano et al., 2017b; Grundy et al., 2019). Recent guidelines recommend a reduction of ≥ 50% in LDL-C from baseline as crucial in very high-risk populations, with a target LDL-C level of <1.4 mmol/L for secondary prevention (Mach et al., 2020). The 2021 AHA/ASA Guideline for the Prevention of Stroke states that for high-risk ischemic stroke patients whose LDL-C levels remain above 70 mg/dL despite receiving maximum tolerated statin therapy combined with ezetimibe, the use of PCSK9 inhibitors for secondary prevention is considered reasonable (Kleindorfer et al., 2021). In our study, the LDL levels at 6 months for the intensive treatment group and the standard treatment group were 1.78 (1.40–2.15) mmol/L (68.83 (54.14–83.14) mg/dL) and 1.13 (0.79–1.37) mmol/L (43.70 (30.55–52.98) mg/dL) (*P* < 0.001), indicating the superiority of adding PCSK9 inhibitors to the treatment over standard lipid-lowering therapy. The FOURIER trial (Sabatine et al., 2017) and ODYSSEY OUTCOMES trial (Schwartz et al., 2018) also demonstrated that PCSK9 inhibitors such as evolocumab and alirocumab, respectively, significantly reduced LDL-C levels and the risk of cardiovascular events compared to statin therapy alone. Emerging evidence indicates that PCSK9 inhibitors not only lower LDL-C but also demonstrate additional lipid-modifying benefits by reducing apolipoprotein B and lipoprotein(a) (Li et al., 2024).

The lipid-lowering effect is not the sole action of PCSK9 inhibitors. They also exhibit anti-atherosclerotic properties, stabilize atherosclerotic plaques, and possess anti-aggregation and anti-coagulation capabilities. Moreover, they demonstrate anti-tumor effects, anti-inflammatory actions, and influence bacterial infection processes in the positive way in adults (Hess, Low Wang & Hiatt, 2018). Treatment with PCSK9 inhibitors has been proven effective in reducing the volume of atherosclerosis (Ako et al., 2019), leading to plaque regression and enhanced stability (Nicholls et al., 2016; Sugizaki et al., 2020). In the GLAGOV clinical trial, a remarkably higher proportion of patients receiving evolocumab exhibited plaque regression compared to the statins group, coupled with a noteworthy decrease in both the percentage of atherosclerotic plaque volume and standardized total atherosclerotic volume (Nicholls et al., 2016). In the initial stages of acute coronary syndrome (ACS), the incorporation of evolocumab with statin therapy may result in incremental enhancement of fibrous-cap thickness and regression of lipid-rich plaques, potentially leading to a more substantial reduction in LDL-C levels (Nicholls et al., 2021; Yano, Horinaka & Ishimitsu, 2020). Additionally, the findings investigating the use of PCSK9 inhibitors during the perioperative period of carotid artery stent placement have highlighted their ability to stabilize carotid artery plaques, diminish perioperative complications, and reduce the recurrence of ischemic events.

Chung et al. (2020) employed HRMRI to evaluate the impact of intensive statin treatment on acute ischemic stroke patients with intracranial atherosclerosis. Their research found that intensive statin therapy effectively reduced and stabilized atherosclerotic plaques. Currently, HRMRI has been conducted to assess the effect of PCSK9 inhibitors on intracranial plaques in ICAS patients undergoing moderate-intensity statin therapy (Li et al., 2023). In a case report, a symptomatic patient with severe stenosis of the middle cerebral artery, who was treated with a combination of high-intensity statins and PCSK9 inhibitors, exhibited a noteworthy decrease in plaque volume, significant improvement in plaque area and stenosis, although no striking difference in vascular remodeling index (Zeng et al., 2023). Another research revealed that, after a 12-week follow-up period, the intensive treatment group displayed notable reductions in stenosis degree compared to the standard treatment group in ICAS patients (Wu et al., 2023). Our finding in the present study among patient with sICAS is consistent with previous studies. We also observed statistically significant alterations in plaque length and volume within the PCSK9 inhibitors group. In addition, the follow-up period of our study was 6 months, which is longer than previous studies.

In high-risk cardiovascular disease patients, the administration of PCSK9 inhibitors demonstrated a noteworthy decrease in the risk of cerebrovascular diseases, encompassing ischemic stroke and recurrent ischemic stroke (Giugliano et al., 2017b; Goodman et al., 2023). In addition, PCSK9 inhibitors has been observed in reducing early recurrent stroke in patients with sICAS during a 1-month follow-up (Wu et al., 2024). In our study, while the intensive treatment group exhibited a notable reduction in recurrent ischemic cerebrovascular events over the 6-month follow-up period, this reduction had no statistical significance, possibly due to the limited sample size. Long-term achievement of lower LDL-C levels is correlated with a decreased risk of cardiovascular and cerebrovascular outcomes, with no apparent safety concerns. The combination of PCSK9 inhibitors with moderate-intensity lipid-lowering therapy has not shown an increased risk of bleeding (Gil-Núñez et al., 2022). The same result was also observed in the ODYSSEY OUTCOMES trial (Jukema et al., 2019). And PCSK9 inhibitors are even the preferred lipid-lowering medications for high-risk patients with hemorrhagic stroke (including patients with a history of hemorrhagic stroke) (Sanz-Cuesta & Saver, 2021). Moreover, the potential impact of PCSK9 inhibitors on cognitive function warrants attention. The FOURIER and ODYSSEY OUTCOMES trials demonstrated that patients treated with PCSK9 inhibitors (alirocumab and evolocumab) showed no significant difference in adverse neurocognitive events compared to placebo (Sabatine et al., 2017; Schwartz et al., 2018). Furthermore, the EBBINGHAUS substudy (part of the FOURIER trial) investigated the effects of very low cholesterol levels on cognitive function in 1,204 patients using the Cambridge Neuropsychological Test Automated Battery (CANTAB) to evaluate memory, processing speed, and executive function. After 19 months of follow-up, the study found no significant cognitive impairment between group (Giugliano et al., 2017a). Additionally, a meta-analysis concluded that PCSK9 inhibitors do not increase neurocognitive risks, even at extremely low LDL-C levels (Robinson et al., 2017). While some studies suggest that statin therapy may modestly elevate diabetes incidence in non-diabetic individuals or worsen glycemic control in patients with type 2 diabetes (T2DM), large-scale clinical trials have consistently demonstrated that PCSK9 inhibitor treatment does not increase diabetes risk (Goodman et al., 2023; Livingstone et al., 2016). Current research indicates the safety, tolerability, and efficacy of PCSK9 inhibitors, without increasing the risk of mortality or adverse neurological events (Blanco-Ruiz et al., 2021; Gaba et al., 2023; Moustafa et al., 2024). These findings align with the outcomes of our study, which revealed no statistically significant differences in adverse events such as cerebral hemorrhage, liver function impairment, myalgia, injection site reaction, gastrointestinal reactions, or risk of new-onset diabetes (*P* > 0.025). In summary, the combined administration of statins and PCSK9 inhibitors enhances lipid-lowering efficacy, addresses gaps in their interactions, and represents a promising direction for the secondary prevention of atherosclerotic diseases in the future.

Limitations of our study include several factors. Firstly, it was not a randomized controlled study, but it is a real-world study that yielded positive results. The small sample size may reduce statistical power, limiting the ability to detect meaningful differences in clinically relevant endpoints, including recurrent ischemic stroke. However, this sample size might still be adequate to detect percentage changes in plaque characteristics between two groups. Larger-scale trials are needed to validate our findings in the future. Moreover, there may be unmeasured confounding factors, such as patient adherence or concomitant medication use, *etc*. Thirdly, the study predominantly focused on narrow arteries in the anterior circulation, but we are also paying attention on the posterior circulation. Fourthly, due to the lack of adipose tissue and relatively small diameter of intracranial arteries, HRMRI imaging constraints hindered a detailed analysis of plaque composition. In addition, our study did not assess the economic implications of PCSK9 inhibitor therapy, which may affect its real-world implementation in resource-constrained settings. Finally, the follow-up period of 6 months is relatively short. The effects of PCSK9 inhibitors on plaque characteristics may vary with longer-term follow-up. Our patients are still under observation and follow-up. Future studies should strive to address these limitations, offering more comprehensive and detailed analyses to enhance the development of clinical diagnosis and treatment strategies, ultimately improving clinical outcomes for patients.

### Conclusion

This study explores the effect of PCSK9 inhibitors on plaque characteristics and the recurrence of ischemic cerebrovascular events in patients with sICAS in the anterior circulation during a 6-month follow-up period. To summarize, PCSK9 inhibitors in combination with statins led to a significant reduction in LDL-C levels and an increased likelihood of reaching LDL-C targets compared to statin monotherapy. Moreover, it showed improvements in arteriosclerosis progression, effectively reducing stenosis degree, plaque length, and volume. Overall, these findings suggest that the use of PCSK9 inhibitors in conjunction with statins is promising for improving the clinical outcomes of stroke patients with sICAS in the future.

### Informed consent

The patients/participants provided their written informed consent to participate in this study.

### Patient and public involvement

Patients or the public were not involved in the design, or conduct, or reporting, or dissemination plans of our research.

## Supplemental Information

10.7717/peerj.20668/supp-1 Supplemental Information 1 Original data

10.7717/peerj.20668/supp-2 Supplemental Information 2 CONSORT checklist

10.7717/peerj.20668/supp-3 Supplemental Information 3 CONSORT flow diagram

10.7717/peerj.20668/supp-4 Supplemental Information 4 Comparative effects of statins monotherapy versus PCSK9 inhibitors combined with statins on LDL reduction and plaque characteristics in patients with sICAS over a 6-month follow-up

10.7717/peerj.20668/supp-5 Supplemental Information 5 Trial protocol

## Additional Information and Declarations

### Data Availability

The following information was supplied regarding data availability: The raw measurements are available in the Supplemental Files.

### Clinical Trial Registration

The following information was supplied regarding Clinical Trial registration: ClinicalTrials.gov NCT05397405. Registered May 23, 2022. https://www.clinicaltrials.gov/ct2/show/NCT05397405.
