# Supplementary figures and images for: Impact of proprotein convertase subtilisin/kexin type 9 (PCSK9) inhibitors on intracranial atherosclerotic plaque characteristics and low density lipoprotein-cholesterol (LDL-C) reduction: a real-world observational study
Source: PeerJ. 2026 Jan 23;14:e20668. doi: 10.7717/peerj.20668 (PMC12834119; doi:10.7717/peerj.20668)

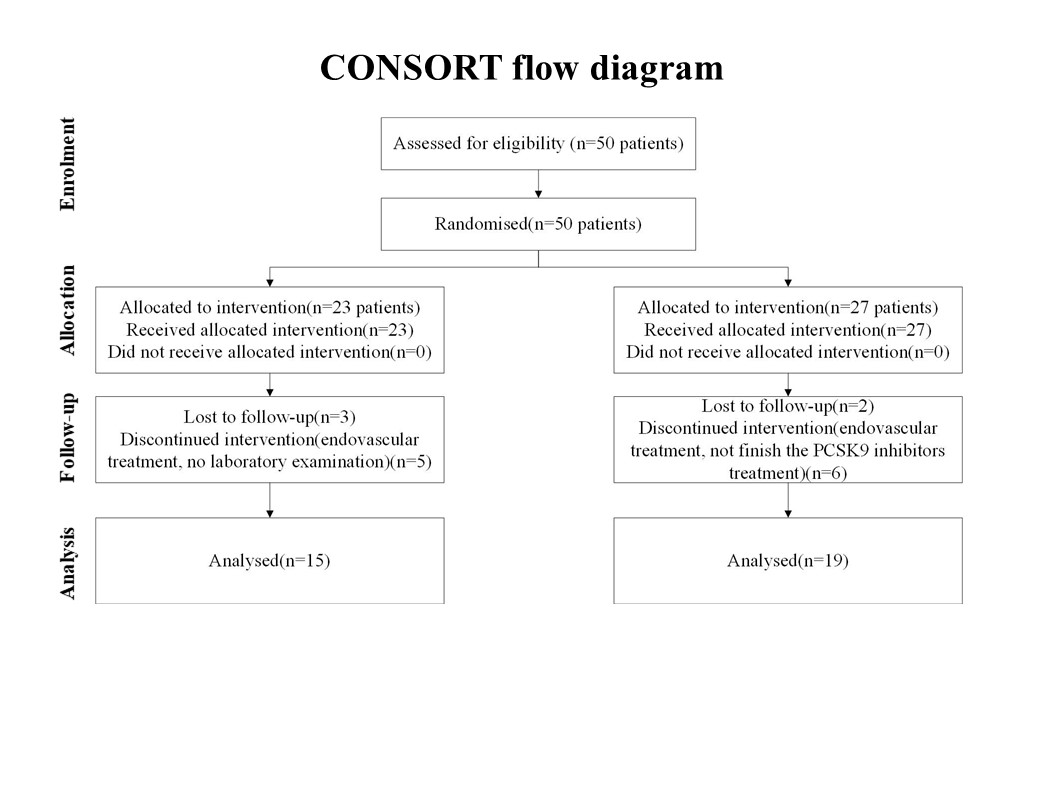

Supplement: Supplemental Information 3 [file peerj-14-20668-s003.jpg]

## Effect of PCSK9 inhibitors on plaque characteristics in patients with sICAS

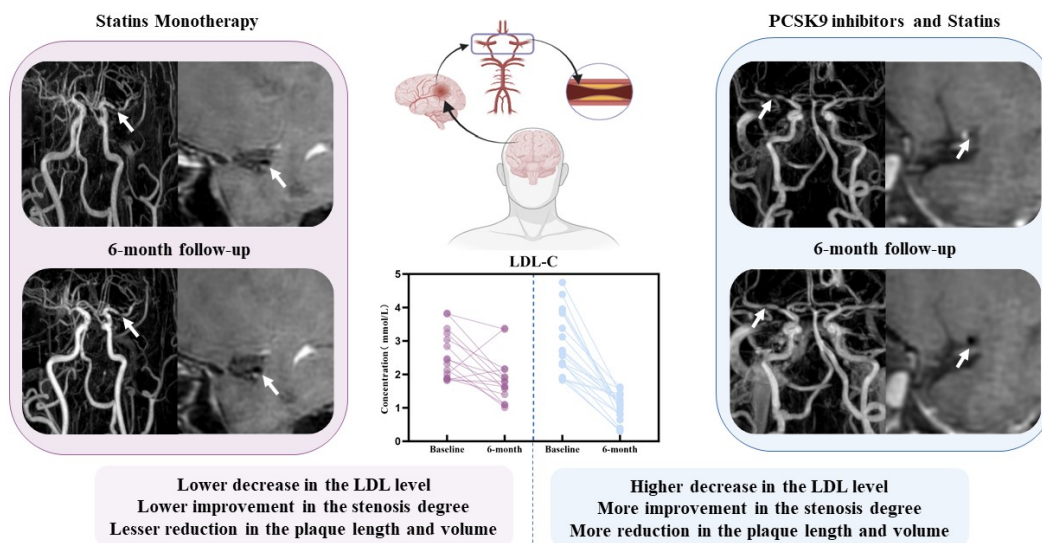

Supplement: Supplemental Information 4 [file peerj-14-20668-s004.pdf]
