# Supplementary material for: Impact of proprotein convertase subtilisin/kexin type 9 (PCSK9) inhibitors on intracranial atherosclerotic plaque characteristics and low density lipoprotein-cholesterol (LDL-C) reduction: a real-world observational study
Source: PeerJ. 2026 Jan 23;14:e20668. doi: 10.7717/peerj.20668 (PMC12834119; doi:10.7717/peerj.20668)
Supplement: Supplemental Information 5 [file peerj-14-20668-s005.pdf]

研究方案

改进血脂管理对症状性颅内动脉狭窄临床预后影响的前瞻性观察性登记研究

研究单位：南京市第一医院

项目负责人：周俊山

承担科室：神经内科

联系电话：02586401149

研究年限：2022 年 03 月－2023 年 07 月

版本号：V1.0

版本日期：2022 年 02 月 27 日

方案摘要

**研究设计**<br>**(可多选)**	□病例对照研究✔队列研究 □横断面研究<br>□随机对照研究 □应用盲法 □其他：
**研究类型（请根据项目类型勾选）**	□伴随诊断试剂盒研究<br>□ 既往临床数据回顾性研究<br>□ 既往临床标本回顾性研究<br>□ 建立标本库研究<br>✔建立队列研究<br>□ I类临床新技术（安全性、有效性确切，技术难度低、几乎不存在伦理风险的医疗技术）<br>□ 既往个案病例报道<br>□ 非植入性医疗器械研究（面罩、牙垫等）<br>□ 已上市 5 年药物研究（包括化药、仿制药等）<br>□ 已上市器械研究（含 AI，影像软件）<br>□ I类临床新技术（安全性、有效性确切，技术难度低、几乎不存在伦理风险的医疗技术）<br>□ 其他（研究者判定，请说明：）
**病例总数**	180
**风险/受益分析**	风险：对于您来说，所有的信息将是保密的。目前相关证据提示脑大动脉粥样硬化改进血脂管理相对安全的，在保证常规基础治疗上改进，全程有专业医师与护士为您保驾护航，如出现不适，由经验丰富的医师为您查找病因，并作出及时方案调整。同时您的样本采集将严格按照无菌要求操作，标本的采集可能会有一些非常小的风险，包括

	短暂的疼痛、局部青紫，少数人会有轻度头晕，或极为罕见的针头感染。受益：通过对您的治疗将有助于评估卒中或需要紧急血运重建的复发性缺血性卒中发生概率，为您的治疗提供必要的建议，并为颅内动脉狭窄血脂管理提供有益的信息。
**风险判断**	✓不大于最小风险□大于最小风险<br>最小风险：指试验中预期风险的可能性和程度不大于日常生活、或进行常规体格检查或心理测试的风险
**研究期限**	2022 年 03 月 01 日至　2023 年 07 月 01 日

一、研究背景

脑卒中是全球第二大常见死因，也是导致永久性后天残疾的主要原因，是我国居民的第一位致残和致死原因且年复发率高达 17.7%[1]。虽然短暂性脑缺血发作不会留下任何损害，但受影响的个体未来发生缺血事件的风险很高，特别是在症状消失后的几天和几周内[2]。症状性颅内动脉粥样硬化性狭窄是指由于动脉粥样硬化导致的颅内动脉狭窄，并在狭窄动脉供血区域发生过缺血性卒中或短暂脑缺血发作，是全球范围内脑卒中的重要病因，尤其在中国和亚洲人群中更为突出[3]。有研究统计，缺血性卒中和短暂性脑缺血发作患者中，颅内动脉狭窄的比例占到 46.6%[4]。对于症状性颅内动脉粥样硬化狭窄 50%以上的缺血性脑卒中或短暂性脑缺血发作的患者先予以标准内科治疗，无效时可血管内介入治疗，但患者的选择应慎重[5]。因此，有效的二级预防是减少复发和死亡的重要手段。血脂异常与动脉粥样硬化密切关联，低密度脂蛋白胆固醇(LDL-C)是公认的动脉粥样硬化性心脑疾病的危险因素，有效的降低 LDL-C 可以降低心脑血管疾病风险[6]。中国缺血性脑卒中和短暂性脑缺血发作二级预防推荐控制 LDL-C 在 1.8mmol/l 以下，已达标准建议 LDL-C 下降幅度 30-50%。虽然我国缺血性卒中患者使用降脂治疗的比例已高达 79.6%，但 LDL-C 达标率仅为 27.4%[7]。有些患者接受最大他汀类药物治疗但仍未达到治疗目标，仍需要额外的降脂治疗。

前蛋白转化酶枯草杆菌蛋白酶/kesin9 型是肝源性分泌蛋白，与 LDLR 的胞外区结合，通过降低肝细胞上 LDLR 的数量，影响 LDL 内化，使血液中 LDL 不能清除，从而提高血浆低密度脂蛋白胆固醇。现有两种全人源 IgG1、IgG2 型单克隆抗体 Alirocumab（阿莫罗布单抗）和 Evolocumab（依洛优单抗）的 PCSK9 抑制剂可用于临床，是一类抑制 PCSK9 的化合物，其主要作用机制是与 PCSK9 分子结合，从而减低 LDL-R 的降解，增加血液中 LDL-C 的吸收来降低其浓度[8]。他汀类药物一直是心脑血管预防降脂治疗的基本支柱[9]，Alirocumab 和 Evolocumab 可将基线 LDL-C 水平降低了约 60%（即使已接受他汀治疗的患者）[10]。降脂作用并不是 PCSK-9 抑制剂具有的唯一作用，如抗动脉粥样硬化作

用和动脉粥样硬化斑块的稳定化、抗聚集作用、抗凝作用、抗肿瘤作用以及影响细菌感染过程的能力[10]，而且在较低的LDL-C未增加出血风险[12]。因此，他汀类药物联合 PCSK9 抑制剂对症状性颅内动脉粥样硬化中重度狭窄对于卒中再发的临床影响和改善预后存在很大的意义[13, 14, 15]。PCSK9 抑制剂在缺血性卒中二级预防中的临床应用专家共识提出对于缺血性卒中合并心肌梗死病史、症状性外周动脉疾病、颅内大动脉粥样硬化性狭窄（狭窄率 70%～99%）或合并多个危险因素的患者，考虑需要长期严格控制 LDL-C<1.8mmol/l。若经最优剂量他汀类未达标，可积极启用 PCSK9 抑制剂（IIb 类推荐，B 级证据）[16]。

颅内动脉包括颅内动脉 C6～7 段、大脑中动脉、大脑前动脉、大脑后动脉、椎动脉 V4 段、基底动脉[17]。颅内动脉中重度狭窄是指以上动脉出现一处或多处狭窄率 50%以上。血管造影是诊断狭窄的金标准，也可通过 MRA、多模 MR 诊断。高分辨磁共振[18、19、20、21、22、23]不仅可以显示血管狭窄程度、斑块大小、性质、负荷、体积、出血、成分、纤维帽厚度、钙化、新生血管、血管壁特征等斑块稳定性指标，对动脉斑块的形态学及组成特征进行评价，有助于判断脑梗死病因及发病机制，进而指导缺血性卒中的临床治疗及预后判断。此外，除动脉粥样硬化外，颅内动脉狭窄的原因还包括烟雾病、原发性中枢系统血管炎及继发性血管炎、纤维肌发育不良等非动脉粥样硬化因素，通过高分辨磁共振可以判断颅内动脉狭窄是否为动脉粥样硬化所致，有助于病因鉴别。

综上所述，目前对于症状性颅内动脉粥样硬化中重度狭窄的血脂管理仍存在以下主要问题：对于改进症状性颅内动脉中重度狭窄的血脂管理目前仍存在以下主要问题：1）对于颅内动脉中重度狭窄的患者改进血脂管理对临床预后是否有效；2）他汀联合 PCSK9 抑制剂治疗的是否存在血管逆重构的可能性尚不明确。结合既往文献报道及前期试验结果，研究旨在探讨他汀联合 PCSK 抑制剂改进对症状性颅内动脉狭窄患者血脂管理是否能降低卒中复发。

参考文献

[1]. 王拥军,王春雪,缪中荣.中国缺血性脑卒中和短暂性脑缺血发作二级预防

指南 2014[J].中华神经科杂志,2015,48(04):258-273.

[2]. 倪金迪,李响,刘梅,等. 脑卒中及短暂性脑缺血发作的二级预防指南核心内容（2014 年 AHA/ASA 版）[J]. 中国临床神经科学,2015(1):65-73..

[3]. 董强,黄家星,黄一宁,徐安定,曾进胜,陈向燕,程忻,范玉华,刘亚杰,孙葳,谭泽峰,徐蔚海.症状性动脉粥样硬化性颅内动脉狭窄中国专家共识[J].中国神经精神疾病杂志,2012,38(03):129-145..

[4]. 中国卒中学会科学声明专家组,濮月华,荆京,宗黎霞.症状性颅内外动脉粥样硬化性大动脉狭窄管理规范——中国卒中学会科学声明[J].中国卒中杂志,2017,12(01):64-71.

[5]. Powers WJ, Rabinstein AA, Ackerson T, Adeoye OM, Bambakidis NC, Becker K, Biller J, Brown M, Demaerschalk BM, Hoh B, Jauch EC, Kidwell CS, Leslie-Mazwi TM, Ovbiagele B, Scott PA, Sheth KN, Southerland AM, Summers DV, Tirschwell DL. Guidelines for the Early Management of Patients With Acute Ischemic Stroke: 2019 Update to the 2018 Guidelines for the Early Management of Acute Ischemic Stroke: A Guideline for Healthcare Professionals From the American Heart Association/American Stroke Association.Stroke.2019Dec;50(12):e344-e418.doi:10.1161/STR.0000000000000211. Epub 2019 Oct 30. Erratum in: Stroke. 2019 Dec;50(12):e440-e441. PMID: 31662037.

[6]. Giugliano RP, Pedersen TR, Park JG, De Ferrari GM, Gaciong ZA, Ceska R, Toth K, Gouni-Berthold I, Lopez-Miranda J, Schiele F, Mach F, Ott BR, Kanevsky E, Pineda AL, Somaratne R, Wasserman SM, Keech AC, Sever PS, Sabatine MS; FOURIER Investigators. Clinical efficacy and safety of achieving very low LDL-cholesterol concentrations with the PCSK9 inhibitor evolocumab: a prespecified secondary analysis of the FOURIER trial. Lancet. 2017 Oct 28;390(10106):1962-1971. doi: 10.1016/S0140-6736(17)32290-0. Epub 2017 Aug 28. PMID: 28859947.

[7]. 王拥军,李子孝,徐安定,刘丽萍,陈康宁.前蛋白转化酶枯草溶菌素 Kexin 9 型抑制剂在缺血性卒中二级预防中的临床应用专家共识[J].中国卒中杂志,2019,14(06):594-599.

[8]. Everett BM, Smith RJ, Hiatt WR. Reducing LDL with PCSK9 Inhibitors--The Clinical Benefit of Lipid Drugs. N Engl J Med. 2015 Oct 22;373(17):1588-91. doi: 10.1056/NEJMp1508120. Epub 2015 Oct 7. PMID: 26444323..

[9]. Tramacere I, Boncoraglio GB, Banzi R, Del Giovane C, Kwag KH, Squizzato A, Moja L. Comparison of statins for secondary prevention in patients with ischemic stroke or transient ischemic attack: a systematic review and network meta-analysis. BMC Med. 2019 Mar 26;17(1):67. doi: 10.1186/s12916-019-1298-5. PMID: 30914063; PMCID: PMC6436237.

[10]. Hess CN, Low Wang CC, Hiatt WR. PCSK9 Inhibitors: Mechanisms of Action, Metabolic Effects, and Clinical Outcomes. Annu Rev Med. 2018 Jan 29;69:133-145. doi: 10.1146/annurev-med-042716-091351. Epub 2017 Nov 2. PMID: 29095667.

[11]. Basiak M, Kosowski M, Cyrnek M, Bułdak Ł, Maligłówka M, Machnik G, Okopień B. Pleiotropic Effects of PCSK-9 Inhibitors. Int J Mol Sci. 2021 Mar 19;22(6):3144. doi: 10.3390/ijms22063144. PMID: 33808697; PMCID: PMC8003510.

[12]. .Gil-Núñez A, Masjuan J, Montaner J, Castellanos M, Segura T, Cardona P, Tembl JI, Purroy F, Arenillas J, Palacio E. Proprotein convertase subtilisin/kexin type 9 inhibitors in secondary prevention of vascular events in patients with stroke: Consensus document and practice guidance. Neurologia (Engl Ed). 2021 Dec 11:S2173-5808(21)00184-X. doi: 10.1016/j.nrleng.2020.11.014. Epub ahead of print. PMID: 34906541.

[13]. Shingai Y, Kimura N, Doijiri R, Takahashi K, Yokosawa M, Kanoke A,

Kikuchi T, Sugawara T, Tominaga T. Effect of Preoperative Administration of Proprotein Convertase Subtilisin/Kexin Type 9 Inhibitor on Carotid Artery Stenting. World Neurosurg. 2020 Mar;135:e36-e42. doi: 10.1016/j.wneu.2019.10.095. Epub 2019 Oct 23. PMID: 31655235.

[14].Aranzulla TC, Musumeci G. Morphological stabilization and regression of carotid plaque following therapy with evolocumab in a high-risk patient. Catheter Cardiovasc Interv. 2021 May 1;97(6):E835-E841. doi: 10.1002/ccd.29231. Epub 2020 Aug 26. PMID: 32845075.

[15].Gupta S. LDL cholesterol, statins and PCSK 9 inhibitors. Indian Heart J. 2015 Sep-Oct;67(5):419-24. doi: 10.1016/j.ihj.2015.05.020. Epub 2015 Aug 5. PMID: 26432726; PMCID: PMC4593843.

[16]. 王拥军,李子孝,徐安定,刘丽萍,陈康宁.前蛋白转化酶枯草溶菌素 Kexin 9 型抑制剂在缺血性卒中二级预防中的临床应用专家共识[J].中国卒中杂志,2019,14(06):594-599.

[17]. [1]中国卒中学会科学声明专家组,濮月华,荆京,宗黎霞.症状性颅内外动脉粥样硬化性大动脉狭窄管理规范——中国卒中学会科学声明[J].中国卒中杂志,2017,12(01):64-71.

[18]. Millon A, Mathevet JL, Boussel L, Faries PL, Fayad ZA, Douek PC, Feugier P. High-resolution magnetic resonance imaging of carotid atherosclerosis identifies vulnerable carotid plaques. J Vasc Surg. 2013 Apr;57(4):1046-1051.e2. doi: 10.1016/j.jvs.2012.10.088. Epub 2013 Feb 1. PMID: 23375613.

[19]. Klein IF, Lavallée PC, Mazighi M, Schouman-Claeys E, Labreuche J, Amarenco P. Basilar artery atherosclerotic plaques in paramedian and lacunar pontine infarctions: a high-resolution MRI study. Stroke. 2010 Jul;41(7):1405-9. doi: 10.1161/STROKEAHA.110.583534. Epub 2010 Jun 10. PMID: 20538696.

[20]. Kim JM, Jung KH, Sohn CH, Moon J, Han MH, Roh JK. Middle cerebral artery plaque and prediction of the infarction pattern. Arch Neurol. 2012 Nov;69(11):1470-5. doi: 10.1001/archneurol.2012.1018. PMID: 22910889.

[21] Mitsumori LM, Hatsukami TS, Ferguson MS, Kerwin WS, Cai J, Yuan C. In vivo accuracy of multisequence MR imaging for identifying unstable fibrous caps in advanced human carotid plaques. J Magn Reson Imaging. 2003 Apr;17(4):410-20. doi: 10.1002/jmri.10264. PMID: 12655579..

[22] Xu WH, Li ML, Gao S, Ni J, Yao M, Zhou LX, Peng B, Feng F, Jin ZY, Cui LY. Middle cerebral artery intraplaque hemorrhage: prevalence and clinical relevance. Ann Neurol. 2012 Feb;71(2):195-8. doi: 10.1002/ana.22626. PMID: 22367991.

[23] Xu WH, Li ML, Gao S, Ni J, Zhou LX, Yao M, Peng B, Feng F, Jin ZY, Cui LY. In vivo high-resolution MR imaging of symptomatic and asymptomatic middle cerebral artery atherosclerotic stenosis. Atherosclerosis. 2010 Oct;212(2):507-11. doi: 10.1016/j.atherosclerosis.2010.06.035. Epub 2010 Jun 25. PMID: 20638663.

二、研究目的

1. 主要目的：对于症状性动脉粥样硬化颅内动脉中重度狭窄的患者，他汀联合使用 PCSK 抑制剂与单独使用他汀治疗在 6-12 个月临床脑卒中复发概率是否有差异。

2. 次要目的：明确研究对以下指标的影响：(1)颅内动脉狭窄程度变化；(2)斑块体积与负荷改变；(3)血管重构指数；(4)缺血组织灌注；(5)评估肌肉相关事件、过敏反应、注射部位反应、出血事件等不良事件的发生率。

三、研究设计、方法与研究步骤

1. 研究设计

采用单中心、前瞻性、观察性登记研究事件的对照、开放标签的、回访者

盲法（PROBE）、基于磁共振检测的对照试验。本研究通过连续入组 160 例符合纳入标准的症状性颅内动脉粥样硬化中重度狭窄、LDL-C>70mg/dl（1.8mmol/L）的患者，观察标准治疗组（阿托伐他汀 20-40mg 或瑞舒伐他汀 10-20mg 或辛伐他汀 20-40mg）80 例与强化治疗组（标准治疗+PCSK9 抑制剂）80 例临床疗效，比较两组间 1、3、6、12 月临床脑卒中复发及影像学指标的差异。该研究将在南京市第一医院完成。样本量计算：预计纳入患者 160 人，平均分组各 80 人，根据样本脱落率 10%，最终需要样本量约为 180 人。本研究旨在探讨他汀联合 PCSK9 抑制剂降脂对症状性动脉粥样硬化颅内动脉中重度狭窄临床预后影响。

2. 研究方法

该研究将采用对比分析法探讨改进血脂管理对症状性颅内动脉粥样硬化中重度狭窄临床预后影响。

3.研究步骤

明确缺血性卒中、短暂性脑缺血发作后，未行溶栓取栓治疗，完善 3T 磁共振血管增强或多模 MR（含高分辨）评估明确血管病变情况以及灌注情况，可行血管造影明确，诊断为症状性动脉粥样硬化颅内动脉中重度狭窄，在保证常规基础治疗稳定病情上，且低密度脂蛋白仍>70mg/dl（1.8mmol/L），予以告知研究方案，并获得知情同意，患者签署入组知情同意书，之后研究者完成各访视点。

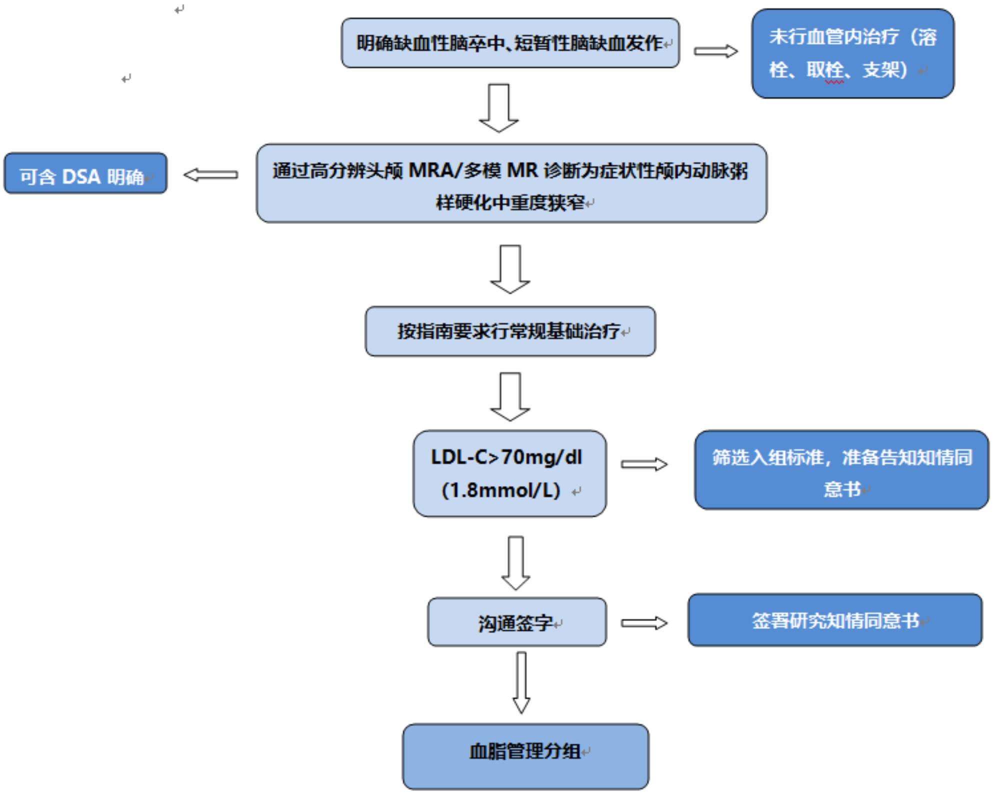

研究方案

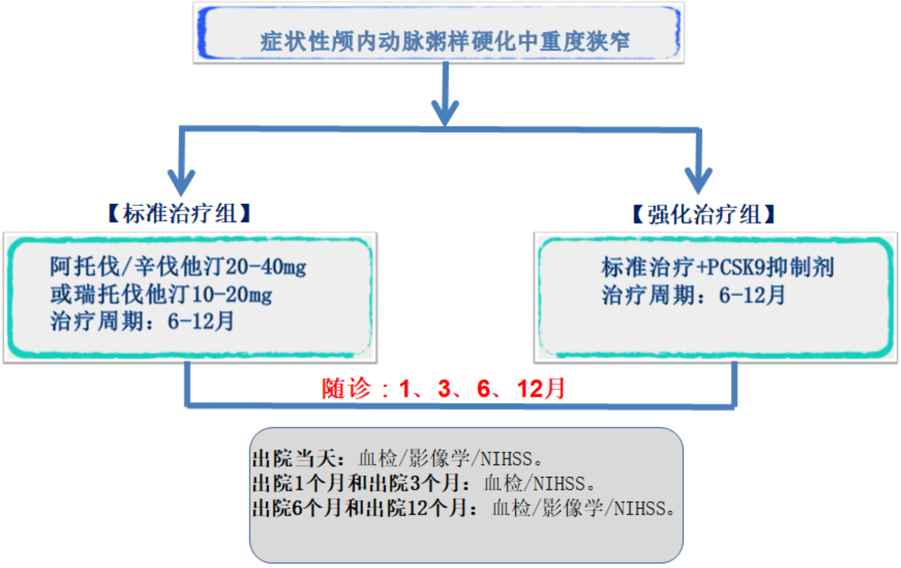

四、病例选择

1. 入选标准

(1) 年龄> 18 岁；

(2) 症状性动脉粥样硬化颅内动脉中重度狭窄，未接受静脉溶栓、取栓治疗以及支架植入等血管内治疗；

(3) 低密度脂蛋白胆固醇>70mg/dl（1.8mmol/L）；

(4) 接受 3T 磁共振血管增强或多模 MR 检查（含高分辨），可含血管造影检查，并获取可供分析图像；

(5) 患者本人或代理人签署知情同意书；

(6) 有他汀药物降脂指征。

2. 排除标准

(1) 他汀类药物禁忌症；

(2) 活动性出血或者已有明显出血倾向者；

(3) 存在严重的心、肺、肾功能不全、恶性肿瘤或其他恶性疾病，预计 7 天内极有可能死亡；

(4) 血管炎、动脉夹层、烟雾病等引起的狭窄；

(5) 存在 MRI 检查禁忌症或不能接受磁共振检查；

(6) 无法控制的严重糖尿病和高血压；

(7) 妊娠。

3. 终止研究标准

研究者需要严格遵守研究方案。但当存在以下情况时，需要停止强化治疗：

(1) 受试者或代理人要求撤回参加本研究的知情同意；

(2) 研究者认为严重不良事件与强化治疗有关；

(3) 研究者认为停止强化治疗可以使受试者更大获益。

(4) 研究者认为其他不适合参与本研究情况。

五、可供选择的其他诊疗方法

目前他汀联合 PCSK 抑制剂对症状性动脉粥样硬化颅内动脉中重度狭窄临床预后影响还未明确，指南推荐 LDL-C 降<1.8mmol/L，有研究表明 PCSK9 抑制剂可有效降低心脑血管风险，PCSK9 抑制剂在缺血性卒中二级预防中的临床应用专家共识提出对于缺血性卒中合并心肌梗死病史、症状性外周动脉疾病、

颅内大动脉粥样硬化性狭窄（狭窄率 70%～99%）或合并多个危险因素的患者，考虑需要长期严格控制 LDL-C<1.8mmol/l。若经最优剂量他汀类未达标，可积极启用 PCSK9 抑制剂（IIb 类推荐，B 级证据）。本研究结合既往文献报告及本课题组的总结发现，他汀联合 PCSK 抑制剂降脂是相对安全的，患者受益程度较前增加，现需进一步行随机对照研究探讨强化治疗症状性动脉粥样硬化颅内动脉中重度狭窄的临床预后影响。

六、检测项目与检测时点

访视 1：出院当天（入组 24 小时）

随访的主要内容是了解患者是否依照分组进行血脂管理以及收集影像学和血检结果，需要收集以下数据：(1)血检报告【血脂指标、大生化全套（含磷脂酶 A2、脂蛋白 a、肝肾功能）、糖化血红蛋白（含平均血糖监测）】；(2)3T 核磁共振血管增强或多模 MR（含高分辨），可含血管造影；(3) NIHSS 评分；(4)既往病史与住院期间的用药及治疗史。

访视 2：出院后 1 个月及访视 3：出院后 3 个月

随访的主要内容是了解患者的短期预后情况。需要收集以下数据：(1)有无卒中或需要紧急血运重建的复发性缺血性卒中发生；(2) 血检报告；(3) NIHSS 评分；(4) 血糖血压监测(5)有无不良事件发生。

访视 4：出院后 6 个月及访视 5：出院后 12 个月

主要内容是了解患者的预后情况。需要收集的数据包括：(1)有无卒中或需要紧急血运重建的复发性缺血性卒中发生；(2)血检报告【血脂指标、大生化全套（含磷脂酶 A2、脂蛋白 a、肝肾功能）、糖化血红蛋白（含平均血糖监测）】；(2)3T 核磁共振血管增强或多模 MR（含高分辨），可含血管造影；(3) NIHSS 评分；(4) 有无不良事件发生；(5) 是否死亡及死亡原因。

七、疗效评定标准

出院当天（入组 24 小时）

随访的主要内容是了解患者是否依照分组进行血脂管理以及收集影像学和

血检结果，需要收集以下数据：(1)血检报告【血脂指标（低密度脂蛋白胆固醇、高密度脂蛋白胆固醇）、磷脂酶 A2、脂蛋白 a、肝肾功能、糖化血红蛋白（含平均血糖监测）】；(2)3T 核磁共振血管增强或多模 MR（含高分辨），可含血管造影；(3) NIHSS 评分；(4)认知功能(5)既往病史与住院期间的用药及治疗史。

出院后 1 个月及出院后 3 个月

随访的主要内容是了解患者的短期预后情况。需要收集以下数据：(1)有无卒中或需要紧急血运重建的复发性缺血性卒中发生；(2) 血检报告；(3) NIHSS 评分；(4) 血糖血压监测(5)有无不良事件发生。

出院后 6 个月及出院后 12 个月

主要内容是了解患者的预后情况。需要收集的数据包括：(1)有无卒中或需要紧急血运重建的复发性缺血性卒中发生；(2)血检报告【血脂指标（低密度脂蛋白胆固醇、高密度脂蛋白胆固醇）、磷脂酶 A2、脂蛋白 a、肝肾功能、糖化血红蛋白（含平均血糖监测）】；(2)3T 核磁共振血管增强或多模 MR（含高分辨），可含血管造影；(3) NIHSS 评分；(4)认知功能；(5) 有无不良事件发生；(6) 是否死亡及死亡原因。

八、不良事件的观察、记录和处置

1 严重不良事件定义

基于国际协调会议指南，根据世界卫生组织国际药物检测中心（WHO-IDMC）1994 年定义，严重不良事件定义为以下任何不良医疗事件：(1) 导致死亡；(2) 主要研究者认为危及生命（在事件发生的时候患者有死亡的危险；不是指假定进一步加重可能会导致死亡的事件）；(3) 需要住院治疗或延长住院时间；(4) 导致持续或有重大意义的残疾或能力障碍；(5) 导致先天性异常或出生缺陷；(6) 主管医师认为重要的医疗事件，不会马上危及生命或导致需要住院，但仍可能危害患者或者需要采取干预措施以预防出现上述任何一种不良事件。

2 记录与报告

使用严重不良事件表记录事件的详细情况，包括对事件的完整描述与分类，以及研究者关于不良事件与血脂干预随机分组的因果关系的意见。所有不良事

件在得知后24小时以内需要尽快向伦理委员会报告，为获得进一步的信息，研究者需要提交跟踪报告以记录不良事件的结果。

3 严重不良事件的监控

伦理委员会需要监控所有的严重不良事件并了解严重不良事件与研究方案的关系。当发现与研究方案相关的严重不良事件过多，需要对研究方案进行修订或提前终止实验，如强化治疗组中出现严重颅内出血事件。

九、研究的质量控制与质量保证

在取得患者知情同意书后，随机患者需要在24小时以内完成。患者入组后，其1个月、3个月、6个月、12个月进行随访。出院后采用门诊或电话联系或登门方式进行随访。随机时将收集患者基本的人口统计学信息与临床数据。随访数据在5个时间点收集：基线、1个月、3个月、6个月、12个月进行随访。数据收集采用统一的患者信息登记表并统一录入数据库中。

十、数据安全监查

本中心（南京市第一医院）具有专门的神经介入导管室与神经影像科，能够满足本研究中血管内治疗与再灌注监测。本中心具有完善的卒中绿色通道，能够保证病例的顺利入组。所有研究者在开展研究工作前均接受专门的培训。本研究由主要研究者组织的监察团队对研究的实施进行监察。首次监察再随机3个患者后进行，其后每月进行一次监察，监察的目的与内容包括：(1) 确认纳入患者的真实性；(2) 确认每个患者知情同意过程无差错；(3) 确认每个患者符合纳入与排除标准；(4) 核查新纳入患者的全部文件；(5) 核查所有完成随访患者的文件。在研究结束后，相关资料与原始文件保存15年以上。

十一、统计学处理

本研究所有数据分析均采用SPASS 20.0，均由南京市第一医院神经内科专职人员完成资料统计。将纳入患者随机分为标准治疗组与强化治疗组，比较两组间基线资料、1个月、3个月、6个月、12个月相应数据差异。主要终点事件6-12个月卒中或需要紧急血运重建的复发性缺血性卒中发生概率比较采用t检

验。其他参数与结局比较中，率的比较实用 Fisher 精确检验，计量资料使用单因素方差分析，有序资料使用 Kruskal-Wallis 检验。

十二、临床研究伦理原则与要求

临床研究将遵循世界医学大会《赫尔辛基宣言》和中华人民共和国国家卫生和计划生育委员会《涉及人的生物医学研究伦理审查办法》等相关规定，具体落实知情同意，保护隐私，研究免费与补偿，控制风险，特殊受试者保护和研究相关损害的赔偿原则与要求。在研究开始之前，由伦理委员会批准该试验方案后才实施临床研究。每一位受试者入选本研究前，研究者有责任向受试者或/和其法定代理人完整、全面地介绍本研究的目的、程序和可能的风险，并签署书面知情同意书，应让受试者知道他们参加临床研究完全是自愿的，他们可以拒绝参加或在试验的任何阶段随时退出本研究而不会受到歧视和报复，其医疗待遇与权益不受影响。知情同意书应作为临床研究文件保留备查，切实保护受试者的个人隐私与数据机密性。

十三、研究进度

2022 年 02 月-2022 年 03 月：临床试验方案批准、可行性报告完成、该项目相关研究者会议、并完成首位受试者入组；

2022 年 03 月-2023 年 03 月：持续受试者入组并完成之前受试者访视，并及时向伦理委员会提交报告；

2023 年 03 月-2023 年 07 月：最后一位受试者完成本研究项目访视，并锁定数据库，进行该研究项目结果报告的撰写。

十四、项目组人员及分工

姓名	职称/专业	任务	签字
周俊山	主任医师	项目主持	
施洪超	主任医师	项目监督	
周峰	副主任医师	受试者入组	

刘宇恺	主治医师	项目协调
邓齐文	住院医师	数据管理与分析
刘成芳	在读研究生	受试者访视
徐肇涵	在读研究生	受试者访视
李晓辉	在读研究生	受试者访视
